# Prediction of hepatic metastasis and relapse in colorectal cancers based on concordance analyses with liver fibrosis scores

**DOI:** 10.1186/s40169-020-0264-3

**Published:** 2020-02-05

**Authors:** Xiang Hu, Audrey Marietta, Wei-Xing Dai, Ya-Qi Li, Xiao-ji Ma, Long Zhang, San-Jun Cai, Jun-Jie Peng

**Affiliations:** 1 Department of Colorectal Surgery, Fudan University Shanghai Cancer Center, 270 Dong’an Road, Shanghai, 20032 China; 2 Department of Oncology, Shanghai Medical College, Fudan University, Shanghai, 200032 China; 3 Universitas Sriwijaya/RSUP Dr. Mohammad Hoesin, Palembang, Indonesia

**Keywords:** Colorectal cancer, Liver metastasis, Liver relapse, Liver fibrosis

## Abstract

**Background:**

Liver fibrosis, resulted from several liver diseases, are increasing up to 25% in population in global. It remains undetermined how much impact liver fibrosis have on the development of hepatic metastasis and relapse in colorectal cancer (CRC). Hence the aim of this study was to clarify the role of liver fibrosis on hepatic metastasis and relapse in CRC undergoing curative therapy.

**Methods:**

We enrolled consecutive 1652 patients with radical colorectal surgery as the discovery cohort, and the validation set enrolled 432 CRC patients with hepatic metastasis. To determine liver fibrosis, the NFS, FIB4 and APRI scores were applied. The influence of liver fibrosis on hepatic metastasis and relapse was assessed by survival analyses. Nomograms with fibrosis score incorporated were established to identify the incremental value for individualized relapse estimation, which was then assessed with respect to calibration, discrimination, and clinical usefulness.

**Results:**

The high liver fibrosis score patients had significantly worse outcomes than low score in 5-year hepatic metastasis (22.6 vs. 8.7%) in discovery cohort, and relapse (58.2 vs. 44.1%) in validation cohort. Multivariate analysis also revealed liver fibrosis as an independent prognostic factor. The distribution analysis also demonstrated higher liver fibrosis score a powerful prognostic factor for hepatic metastasis and relapse. The nomogram incorporated with liver fibrosis score resulted in better performance than TNM staging system and clinicopathologic nomograms. Importantly, the discriminatory capacity of the fibrosis score was superior to that of the CRS score in predicting hepatic specific disease-free survival (DFS) and relapse-free survival (RFS), as demonstrated by the C-index and AUC. The concordance study showed well agreement among NFS, FIB4 and APRI in predicting DFS and RFS. Among these three noninvasive liver fibrosis scores, NFS score performed the best in predicting hepatic specific DFS and RFS.

**Conclusion:**

The liver fibrosis was a powerful predictor of hepatic specific DFS and RFS in CRC. Fibrosis niche may be a favorable microenvironment for metastatic formation in the liver.

## Background

The liver is the leading site for metastases for colorectal cancer (CRC). About 20% of patients with CRC occurred synchronous hepatic metastasis at the time of diagnosis, what is more, another 50% of patients with radical surgery will go on to develop metachronous hepatic metastasis during the follow-ups [1, 2]. Therefore, there is an urgent need for an accurate classification predicting hepatic metastasis and relapse. The current commonly used prognostic model for risk classification relies mainly on the TNM staging system and CRS score [3]. However, great variations in clinical outcomes have been demonstrated in patients even with the same stage. Hence, limited consensus was achieved regarding the relative importance of the reported prognostic factors [4].

Non-alcoholic fatty liver disease (NAFLD), non-alcoholic steatohepatitis (NASH) and hepatotropic co-infections (due to hepatitis B and hepatitis C virus (HBV, HCV)) are prevailed liver diseases, which affects up to 25% of western population [5]. And then those patients would develop liver fibrosis at last. Given the high occurrence of liver fibrosis, the association between liver metastasis in CRC and liver fibrosis is considered to be interesting area. However, there existed controversy in terms of the association between liver metastasis and liver steatosis (early stage of liver fibrosis) [6, 7]. Thus, the protective or promoting effect of liver fibrosis on the liver metastasis in CRC remains to be completely elucidated.

Presently, noninvasive markers of liver fibrosis are available including NFS, FIB4 and APRI scores. So, these preoperatively noninvasive liver fibrosis scores were supposed to facilitate stratification of different populations, provided individualized prognostic data and improved outcomes. Therefore, the main aim of this study was to estimate predictors of hepatic metastasis and relapse in CRC as evaluated by NFS, FIB4 and APRI scores. Moreover, the concordance among these three scores was assessed to validate the accuracy.

## Methods

### Patient population

This study included two independent cohorts of CRC patients. Under approval from the Fudan University Shanghai Cancer Center (FUSCC) review board, we enrolled consecutive 1652 patients with CRC radical surgery from the colorectal cancer prospective surgery dataset from 2012 to 2013 as the discovery cohort. Our inclusion criteria for this discovery cohort analysis consisted of [1] histologically confirmed colorectal cancer [2] underwent radical surgery. Our exclusion criteria were [1] stage IV disease, [2] inappropriate data for calculating the NFS, FIB4 and APRI preoperatively and [3] death within 1 month postoperatively.

The validation set enrolled 432 CRC patients with hepatic metastasis treatment from 2014 to 2016. The criteria for this validation cohort analysis included [1] stage IV disease with only liver metastasis from colorectal cancer [2] underwent radical therapy (surgery or ablation). Our exclusion criteria were [1] concomitant other organ metastasis, [2] inappropriate data for calculating the NFS, FIB4 and APRI preoperatively and [3] death within 1 month postoperatively.

### Data characteristics

Age at the diagnosis, race, tumor localization, diagnostic year and other demographic data were obtained from hospital records. Laboratory data collected before the operation included alanine aminotransferase (ALT), aspartate aminotransferase (AST), albumin levels, blood routine, and fasting glucose level. The concomitant diabetes mellitus (DM) or use of antidiabetic drugs and obesity (a body mass index > 25 kg/m^2^) were also recorded.

### Calculation of liver fibrosis score

To determine the degree of liver fibrosis, the NFS, FIB4 and APRI were applied in this analysis. The NFS score [8] was calculated as follows: NFS = − 1.675 + 0.037 × age (years) + 0.094 × BMI (kg/m^2^) + 1.13 × IFG (impaired fasting glucose)/diabetes (yes = 1, no = 0) + 0.99 × AST/ALTratio-0.013 × platelet count (10^9^/L) − 0.66 × albumin level (g/dL). The FIB4 score was calculated according to Sterling’s formula [9], as follows: age (years) × AST (IU/L)/platelet count (10^9^/L) × (ALT^1/2^(IU/L). The APRI score was calculated as Wai’s formula [10]: (AST/platelet count (10^9^/L) × 100. The liver fibrosis was ranked as follows: NFS low, < 0.676; NFS high, > 0.676; FIB4 low, < 1.45; FIB-4 intermediate, from 1.46 through 3.25; FIB-4 high, > 3.25; APRI low, < 0.5; APRI high, > 0.51.

### Evaluation of incremental value of liver fibrosis score in individual survival estimation

To determine the incremental value of the liver fibrosis score in individual DFS and OS estimation, both a liver fibrosis nomogram and a clinical nomogram were established in the discovery and validation cohorts. The liver fibrosis nomogram incorporated the liver fibrosis score and the prognostic clinical risk factors. The clinical nomogram included only the prognostic clinical risk factors. Calibration discrimination (C-index), reclassification, and area under curve (AUC) were used to assess the incremental value of the liver fibrosis score to the TNM staging system and other clinical risk factors. Then the performance of those nomograms was compared.

### Statistical analysis

Continuous variables were presented as mean ± standard deviation. Categorical variables were shown as the number of cases and percentages. The Mann–Whitney U-test Comparisons were performed for continuous variables comparison, The X^2^-test or Fisher’s exact test were used for binary variables. Kaplan–Meier curves and multivariate Cox proportional hazards regression were used to determine any significant difference between curves for survival outcomes. To assess the potential effect modification by age, sex, BMI, and DM, the interaction analysis was utilized by comparing the respective categorical variables in sub-group. All statistical tests were two-sided, and P-values less than 0.05 were considered to be statistically significant. Nomograms and calibration plots were generated using the rms package of R software [11]. Then calibration plots and Receiver Operating Characteristic were performed to investigate the performance of nomograms. All other statistical analyses were performed using IBM SPSS statistics Version 22 (SPSS Inc.; IBM Corporation Software Group, Somers, NY, USA).

## Results

### Characteristics of the consecutive cases with newly diagnosed colorectal cancer

During the 2-year study period from 2012 to 2013, 1652 patients with CRC radical surgery were included in the hepatic metastasis cohort. The baseline clinicopathological parameters of patients were listed in Table 1. The mean follow-up period was 68 ± 23 months. Three noninvasive markers of liver fibrosis were identified, including the non-alcoholic fatty liver disease fibrosis score (NFS), FIB4 score and the aspartate aminotransferase-to-platelet ratio index (APRI). The mean NFS, FIB4 and APRI were determined to be − 1.94 ± 1.33, 1.24 ± 0.72, and 0.23 ± 0.188. Based on the original formula, 29 (1.9%)–89(5.4%) patients were diagnosed as having fibrotic liver with advanced fibrosis score according to varied score system.

**Table 1:** Baseline characteristics of the consecutive CRC patients with radical surgery

Variables, N (%)	CRC (N = 1652)
Gender	
Male	982
Female	670
Age, years	58.7 ± 12.1
TNM stage	
I	479
II	623
III	550
T stage	
T1	217
T2	513
T3	186
T4	736
N stage	
N0	1101
N1	369
N2	182
Location	
Right-sided colon	338
Left-sided colon	318
Rectum	996
Histological type	
Adenocarcinoma	1529
Mucinous	123
Lymph node examined	
Median	16 ± 6
Perineural invasion	
Negative	1424
Positive	228
Vascular invasion	
Negative	1348
Positive	304
Adjuvant chemotherapy	
No	675
Yes	977
Diabetes mellitus	168
Obesity (BMI > 25)	423

### Hepatic metastasis occurrence postoperatively and liver fibrosis

During the mean follow-up period of 68 months. hepatic metastasis was observed in 148 (8.9%) of 1652 patients. According to the original formula, hepatic metastases were observed in 141 (8.7%) of 1621 patients in the low liver fibrosis score group, while in 7 (22.6%) of 31 in the high liver fibrosis score group based on NFS. And according to FIB4, only 8.1% patients in the normal liver group developed hepatic metastasis, but 27.6% patients with high fibrosis score had hepatic metastasis. All the same, patients with high fibrosis score in APRI also occurred more hepatic metastases than those with low fibrosis score (14.6% vs. 8.6%). In all, they indicated a significant difference hepatic metastasis (all P < 0.05, see Table 2).

**Table 2:** Prevalence of any hepatic metastasis in cohorts stratified by NFS, FIB4 and ARPI

Group (%)	Hepatic metastasis free	Hepatic metastasis	P value
NFS			
Low	1480 (91.3)	141 (8.7)	0.017
High	24 (77.4)	7 (22.6)	
NFS modified			
Low	1009 (92.6)	81 (7.4)	0.003
High	495 (88.1)	67 (11.9)	
FIB4			
Low	1112 (91.9)	98 (8.1)	0.001
Intermediate	371 (89.8)	42 (10.2)	
High	21 (72.4)	8 (27.6)	
FIB4 modified			0.018
Low	1112 (91.9)	98 (8.1)	
Intermediate	336 (89.8)	38 (10.2)	
High	56 (82.4)	12 (17.6)	
APRI			0.049
Low	1428 (91.4)	135 (8.6)	
High	76 (85.4)	13 (14.6)	

### Survivals and liver fibrosis

**Fig. 1:**
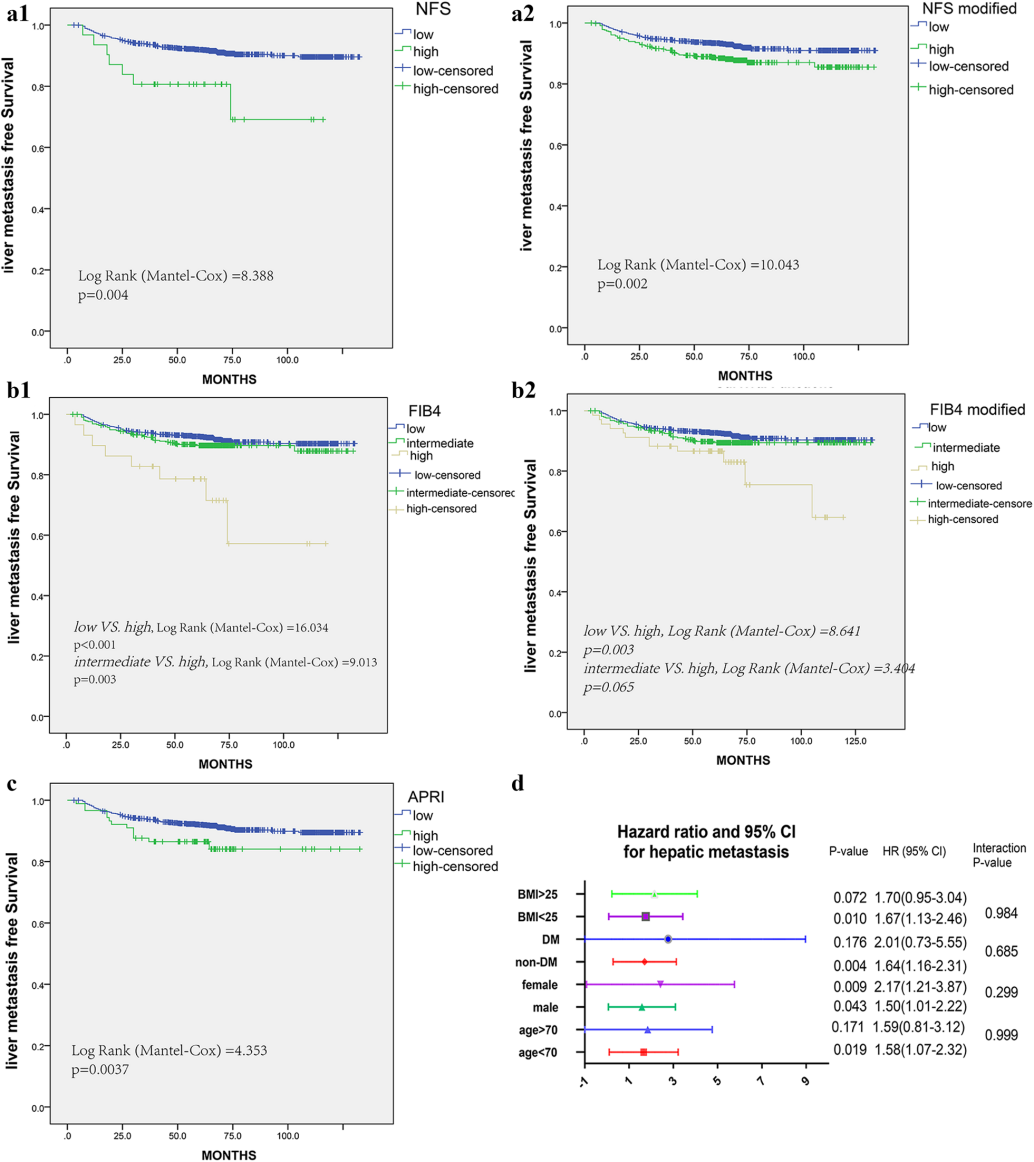
Liver fibrosis score in NFS, FIB4, APRI measured by Kaplan–Meier survival for DFS in the discovery cohort. **a1** original NFS score, **b2** modified NFS score, **b1** original FIB4 score, **b2** modified FIB4 score; **c** APRI score; **d** Forest plot of the subgroup analysis showing no effect modification evident by age, gender, BMI, and DM

The hepatic metastasis-free survival (DFS) and Over-all survival (OS) are shown in Fig. 1 (a1, b1) and Additional file 1: Figure S1 (A1, B1). Patients with high liver fibrosis scores had significantly worse survival rates (all P < 0.05), no matter NFS or FIB4 classification. All the same, high score classified by APRI had a significantly shorter DFS (P = 0.0037, Fig. 1c), while no significance for OS (P = 0.079, Additional file 1: Figure S1C).

What is more, the X-tile plot was performed to achieve the optimum cutoff value for NFS, FIB4 and APRI scores (− 1.36, 2.6, and 0.5, respectively). The hepatic metastasis-free survivals were compared in modified score groups using Kaplan–Meier analysis, see Fig. 1 (a2, b2). All the same, the low fibrosis group had better 5-year DFS rate than the high fibrosis group (NFS modified: 93.2 versus 88.4%; P = 0.002; FIB4 modified: 92.6 versus 89.4%, P = 0.003). Regarding to the OS, the low fibrosis group also indicated better 5-year survival rate (NFS modified: 93.3 versus 85.1%; P < 0.001; FIB4 modified: 92.4 versus 79.1%; P = 0.006), see Additional file 1: Figure S1 (A2, B2). In addition, hepatic metastases were also observed much more in patients with high score, indicating a significant difference (all P < 0.05, see Table 2).

Univariate and multivariate analyses for hepatic DFS and OS were presented in Tables 3 and 4. These results demonstrated that liver fibrosis was a significantly prognostic factor for liver metastasis and OS. The HR for hepatic specific DFS in subgroup analysis indicated that the patients’ backgrounds might also mattered (Fig. 1d). However, the interaction analysis suggested no effect modification by age, gender, BMI, and DM, with overall P-values of 0.999, 0.299, 0.52, 0.984, and 0.685, respectively.

**Table 3:** Risk factors for any hepatic metastasis postoperatively

Variables	Univariate analysis			Multivariate analysis		
	Hazard ratio	95% CI	P value	Hazard ratio	95% CI	P value
Liver fibrosis score						
NFS	1.677	1.213–2.319	0.002	1.664	1.189–2.329	0.003
FIB4	1.439	1.109–1.867	0.006	1.453	1.106–1.909	0.007
APRI	1.816	1.028–3.209	0.04	2.34	1.311–4.176	0.004
Male gender	1.555	1.098–2.203	0.013	1.49	1.049–2.116	0.026
Age > 70	1.508	1.043–2.181	0.029	1.521	1.031–2.244	0.034
T stage						
T1	Reference			Reference		
T2	1.536	0.735–3.210	0.254	1.456	0.696–3.046	0.319
T3	2.416	1.086–5.379	0.031	1.789	0.788–4.064	0.165
T4	2.945	1.483–5.848	0.002	2.168	1.080–4.352	0.03
N stage						
N0	Reference					
N1	1.456	1.012–2.094	0.043	0.174	0.893–1.862	0.174
N2	1.022	0.592–1.765	0.938	0.981	0.581–1.744	0.981
Location						
Right-sided colon	Reference			Reference		
Left-sided colon	0.616	0.383–0.991	0.046	0.702	0.434–1.138	0.151
Rectum	0.546	0.378–0.788	0.001	0.749	0.496–1.130	0.168
Lymph node examined	1.017	0.993–1.041	0.157			
Mucinous Adenocarcinoma	1.756	1.073–2.876	0.025	1.39	0.839–2.304	0.202
Perineural invasion	1.298	0.845–1.996	0.234			
Vascular invasion	1.333	0.909–1.954	0.142			
Adjuvant therapy	2.983	1.987–4.476	< 0.001	2.726	1.782–4.171	< 0.001
Diabetes mellitus	0.996	0.584–1.700	0.99			
Obesity (BMI > 25)	1.292	0.913–1.831	0.149

**Table 4:** Risk factors for over-all survival

Variables	Univariate analysis			Multivariate analysis		
	Hazard ratio	95% CI	P value	Hazard ratio	95% CI	P value
Liver fibrosis score						
NFS	1.772	1.307–2.403	< 0.001	1.683	1.22–2.312	0.001
FIB4	1.487	1.165–1.899	0.001	1.453	1.12–1.878	0.004
APRI	1.652	0.938–2.912	0.082			
Male gender	1.57	1.132–2.179	0.007	1.518	1.09–2.113	0.013
Age > 70	1.834	1.315–2.560	< 0.001	1.784	1.25–2.540	0.01
T stage						
T1	Reference			Reference		
T2	1.502	0.77–2.927	0.233	1.471	0.75–2.870	0.258
T3	2.025	0.956–4.288	0.065	1.571	0.72–3.391	0.25
T4	2.647	1.419–4.938	0.002	2.012	1.06–3.792	0.031
N stage						
N0	Reference			Reference		
N1	1.446	1.026–2.038	0.035	1.287	0.9–1.821	0.153
N2	1.017	0.608–1.699	0.95	1.016	0.60–1.703	0.951
Location						
Right-sided colon	Reference			Reference		
Left-sided colon	0.542	0.343–0.856	0.009	0.044	0.39–0.988	0.151
Rectum	0.526	0.374–0.740	< 0.001	0.718	0.48–1.053	0.09
Lymph node examined	1.369	0.946–1.981	0.096			
Mucinous Adenocarcinoma	1.667	1.034–2.688	0.036	1.323	0.81–2.161	0.264
Perineural invasion	1.169	0.76–1.776	0.464			
Vascular invasion	1.367	0.95–1.956	0.088			
Adjuvant therapy	2.418	1.68–3.461	< 0.001	2.301	1.57–3.358	< 0.001
Diabetes mellitus	1.003	0.60–1.656	0.991			
Obesity (BMI > 25)	1.296	0.93–1.799	0.12			

Meanwhile, we also performed survival analyses for Perineural invasion, Vascular invasion, Histological type and TNM stage system (Additional file 1: Figures S2 and S3).

We also evaluated the distribution of liver fibrosis score, hepatic metastasis and survival statuses (Additional file 1: Figure S4). Higher liver fibrosis scores remained a powerful and independent prognostic factor for hepatic metastasis and dead.

To further identify whether liver fibrosis score could stratify patients by TNM stage, we assessed the prognostic role of liver fibrosis score in patients with stage I + II and stage III subgroups (Additional file 1: Figure S5). It turned out that patients with high liver fibrosis scores still had significantly worse hepatic DFS than those with low scores, no matter stage I + II or stage III disease.

### Incremental value of liver fibrosis score in individual hepatic DFS performance

**Fig. 2:**
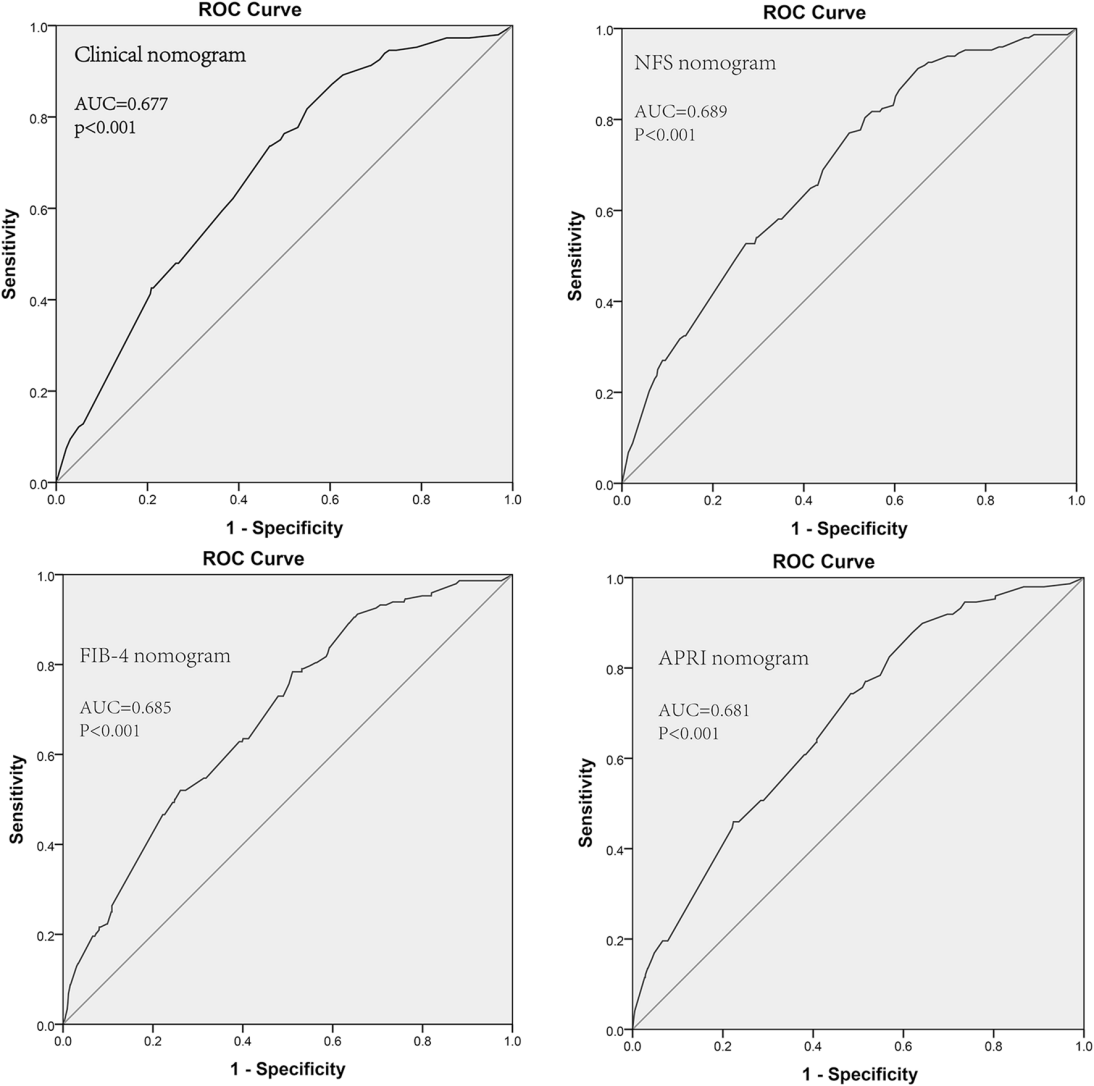
Liver fibrosis score nomogram measured by ROC curves for DFS in the discovery cohort. *AUC* area under the curve, *ROC* receiver operator characteristic. Among NFS, FIB4 and APRI, NFS did the best performance

**Fig. 3:**
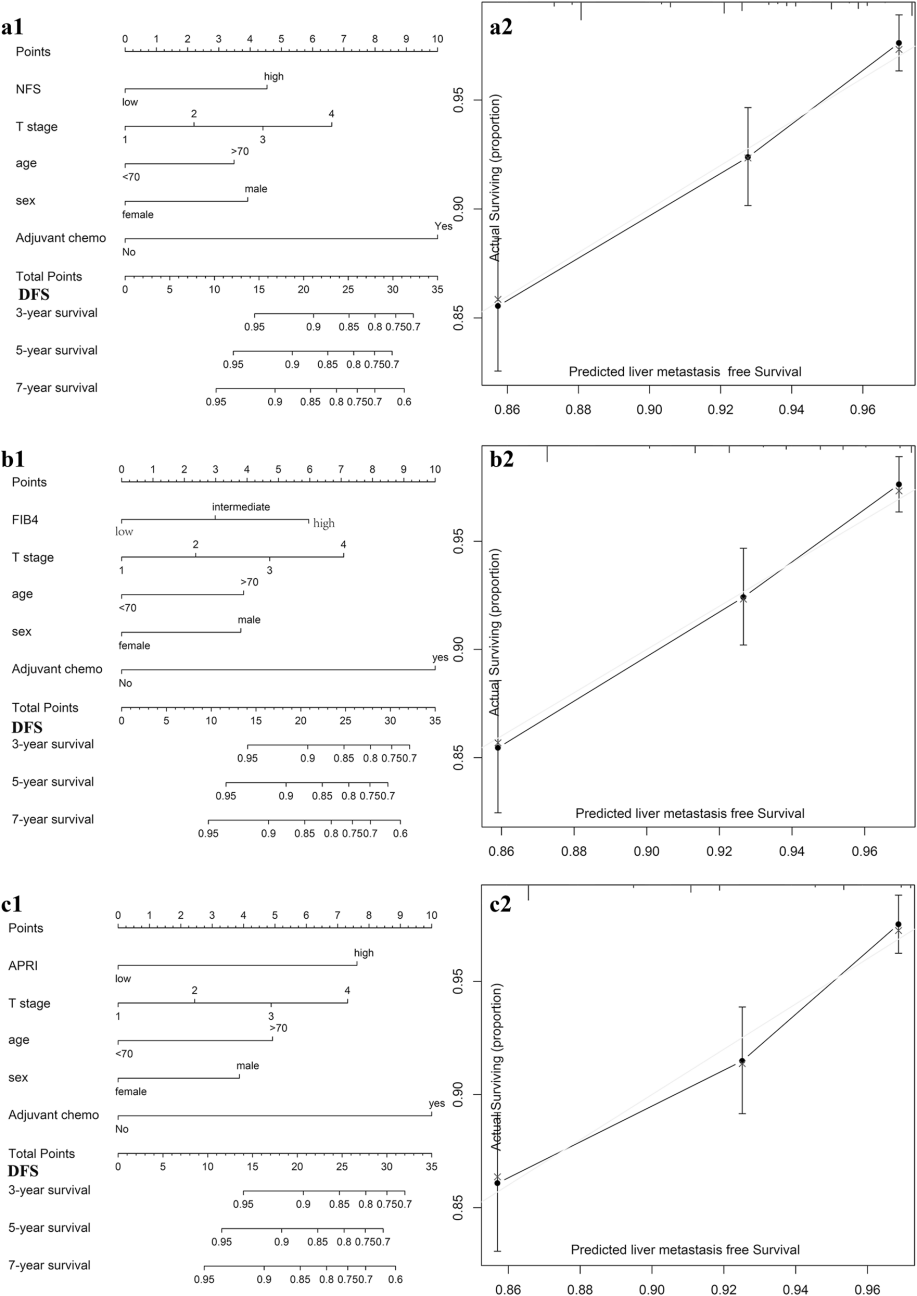
Construction of liver fibrosis score nomogram to estimate hepatic DFS in the discovery cohort, along with the assessment of the model calibration. **a1** NFS nomogram, **a2** Calibration curves for NFS. **b1** FIB4 nomogram, **b2** Calibration curves for FIB4; **c1** APRI nomogram, **c2** Calibration curves for APRI

We aimed to offer a quantitative way to predict the probability of time dependent hepatic DFS, and to identify the incremental value of liver fibrosis score to the TNM stage system for individualized assessment of DFS. Hence, both the liver fibrosis nomogram and clinicopathologic nomogram were established. Four clinicopathologic risk factors in the models: TNM stage, gender, age, adjuvant chemotherapy, were all significantly associated with DFS. The clinicopathologic nomograms for DFS and OS using these clinicopathologic risk factors were established (Additional file 1: Figure S7). C-index, and AUC estimates for the different models showed in Fig. 2. The liver fibrosis nomograms for DFS (Fig. 3) and OS (Additional file 1: Figure S6) were plotted. And the calibration curves of the nomograms showed good agreement between the estimations and actual observations. Compared to either the TNM stage or the clinicopathologic nomogram, the liver fibrosis nomogram showed a better discrimination capability in DFS and OS. The corresponding prediction error curves of models, in Additional file 1: Figure S8, showed that the liver fibrosis nomogram had a faintly lower error than the clinicopathologic nomogram and the TNM. The C-indexes and AUC of the liver fibrosis nomogram predicting DFS and OS were higher than those of TNM stage and clinicopathologic risk factors. Among them, NFS did the best performance.

In addition, we also generated nomograms with only conventional clinical metrics (Perineural invasion, Vascular invasion, Histological type and so on) and liver fibrosis score with clinical features, for DFS and OS, respectively (Additional file 1: Figures S9). We also found that the combination of clinical conventional metrics did not improve the classification results even more compared with the liver fibrosis nomogram (C-index: DFS 0.663 vs. 0.686; OS 0.663 vs. 0.695).

### Characteristics of the liver metastatic CRC

**Fig. 4:**
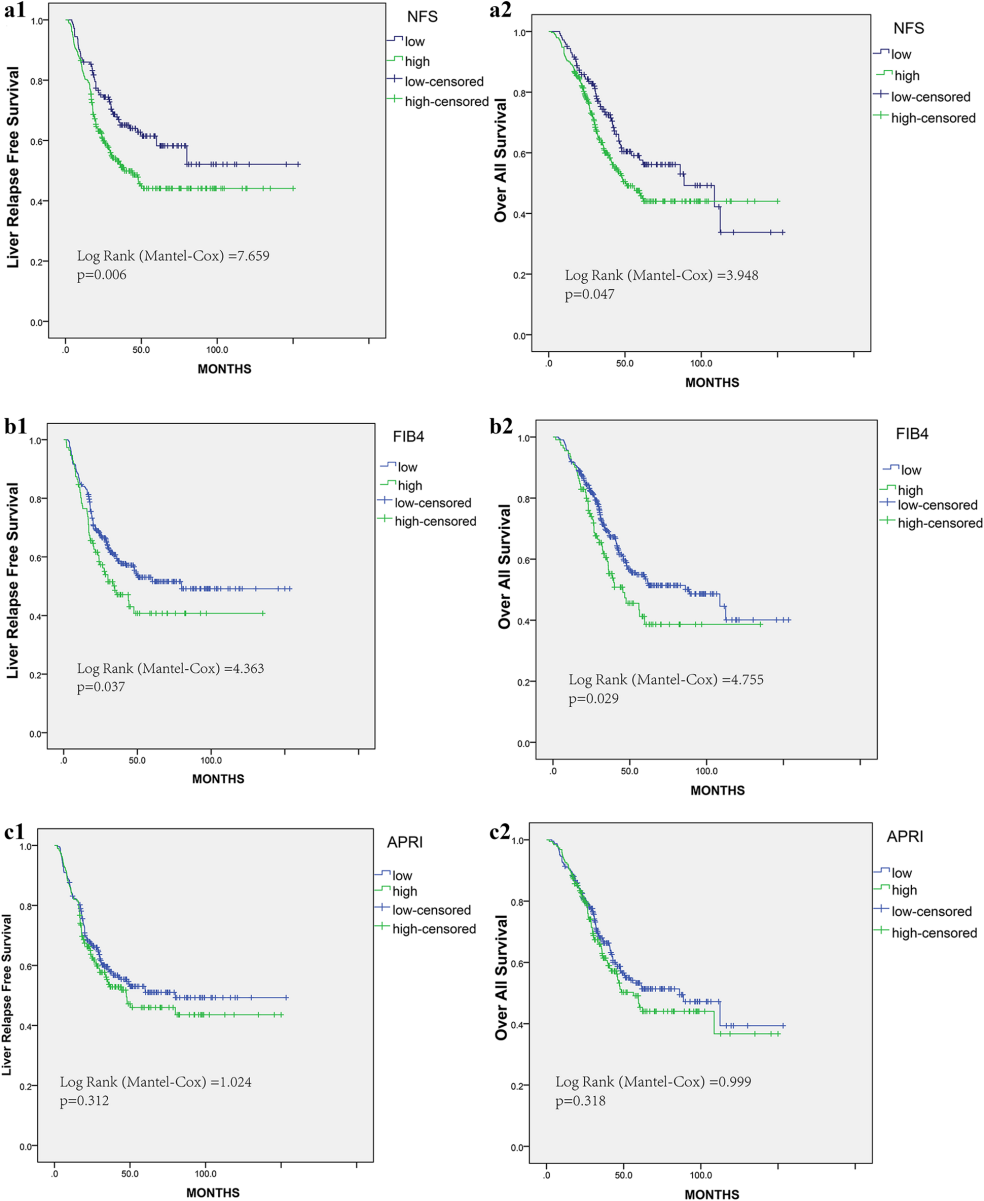
Liver fibrosis score in NFS, FIB4, APRI measured by Kaplan–Meier survival for liver RFS and OS in the validation cohort. **a1** NFS for liver RFS, **a2** NFS for OS. **b1** FIB4 for liver RFS, **b2** FIB4 for OS. **c1** APRI for liver RFS, **c2** APRI for OS

From 2014 to 2016, 432 CRC patients with hepatic metastasis were included in the hepatic relapse cohort. The baseline clinicopathological parameters of patients were listed in Table 5. The mean follow-up period was 42 ± 27 months. The three noninvasive markers of liver fibrosis: NFS, FIB4 and APRI were calculated. The mean NFS, FIB4 and APRI were determined to be − 2.19 ± 1.5, 1.16 ± 0.68, and 0.15 ± 0.1. The optimum cutoff values achieved by X-tile plots were − 2.65, 1.37 and 0.13, respectively. The hepatic relapse-free survival (RFS) and Over-all survival (OS) analyses were presented in Fig. 4. Patients with high liver fibrosis score had significantly worse survival rates (all P < 0.05), no matter NFS or FIB4 classification. In detail, the high fibrosis score group had worse 5-year RFS rate than the low fibrosis score group (NFS: 44.1 versus 58.2%; P = 0.006; FIB4: 40.7 versus 51.6%, P = 0.037). Regarding to the OS, the high fibrosis group also indicated shorter 5-year OS rate (NFS: 45.8 versus 57.6%; P = 0.047; FIB4: 38.6 versus 53.5%; P = 0.029). In addition, liver fibrosis score classified by APRI was not significantly associated with RFS and OS (P = 0.312 and P = 0.318).

**Table 5:** Baseline characteristics of the CRC patients with liver metastasis

Variables, N (%)	mCRC (N = 432)
Synchronous metastasis	
Yes	271
No	161
Gender	
Male	237
Female	195
Age, years	57.6 ± 11.3
TNM stage	
I	14
II	48
III	99
IV	271
T stage	
T1	13
T2	39
T3	132
T4	248
N stage	
N0	161
N1	172
N2	99
CRS	
≤ 2	237
> 2	195
Size of largest liver metastasis (cm)	3.05 ± 1.8
No. of liver metastasis	3 ± 2
Neo-adjuvant chemotherapy	246
Location	
Right-sided colon	109
Left-sided colon	114
Rectum	209
Histological type	
Adenocarcinoma	414
Mucinous	18
Lymph node examined	
Median	15 ± 7
Perineural invasion	
Negative	308
Positive	124
Vascular invasion	
Negative	309
Positive	123
Therapy	
Surgery	305
Ablation	127
Diabetes mellitus	43
Obesity (BMI > 25)	88
CEA (ng/mL)	40.7 ± 95

**Fig. 5:**
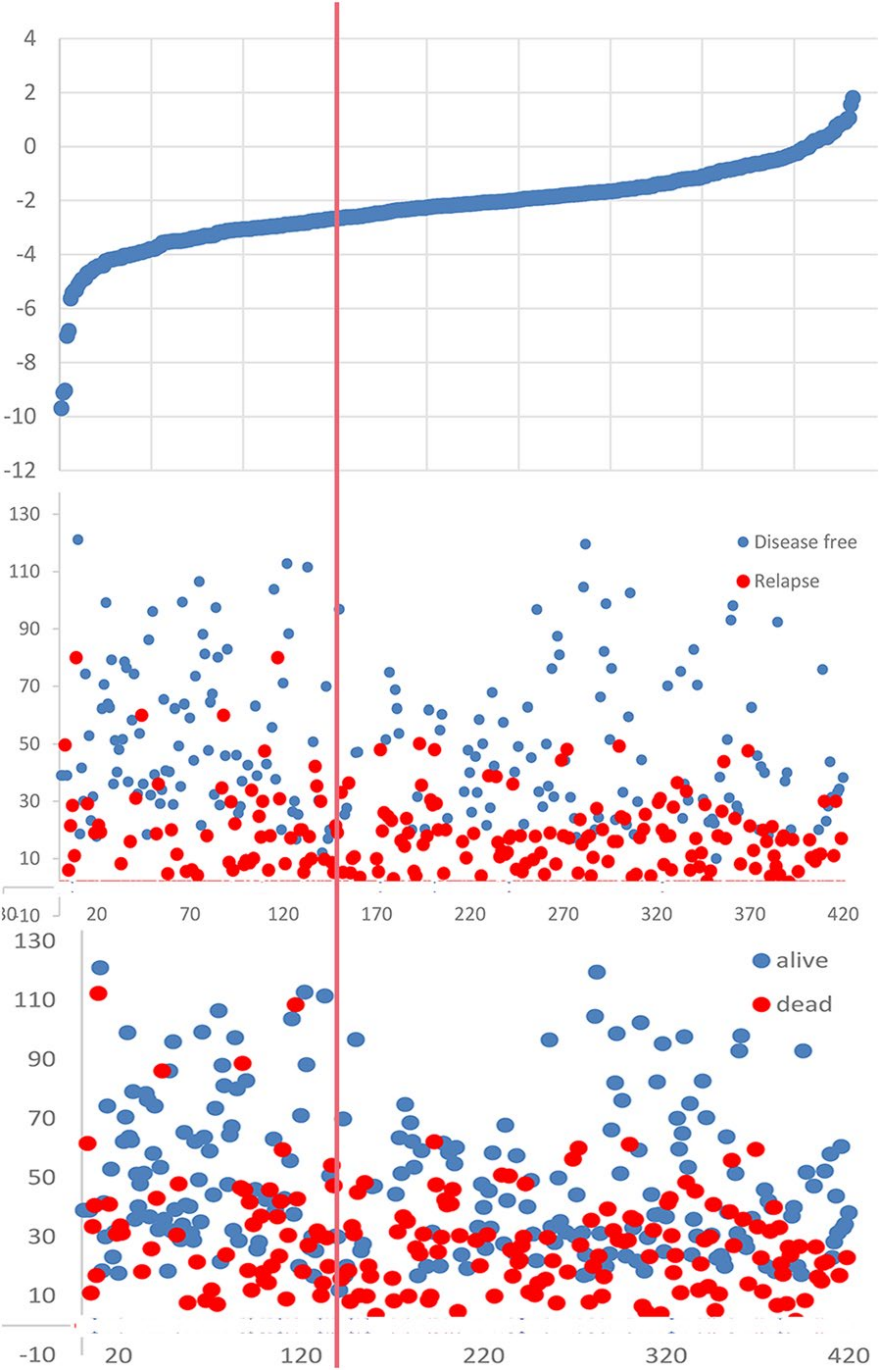
Distribution of liver fibrosis score, hepatic relapse and survival statuses in validation cohort. Higher liver fibrosis score remained a powerful and independent prognostic factor for hepatic relapse and dead

We also evaluated the distribution of liver fibrosis score, hepatic relapse and survival statuses (Fig. 5). Higher liver fibrosis score remained a powerful and independent prognostic factor for hepatic relapse and dead.

Univariate and multivariate analyses for hepatic RFS were presented in Table 6. These results demonstrated that liver fibrosis was also a significantly prognostic factor for hepatic RFS.

**Table 6:** Risk factors for liver relapse postoperatively

Variables	Univariate analysis			Multivariate analysis		
	Hazard ratio	95% CI	P value	Hazard ratio	95% CI	P value
Liver fibrosis score						
NFS	2.42	1.565–3.743	< 0.001	1.488	1.086–2.039	0.013
FIB-4	1.387	1.018–1.890	0.038	1.368	1.004–1.864	0.047
APRI	1.156	0.872–1.532	0.313			
CRS						
≤ 2	Reference			Reference		
> 2	1.995	1.502–2.651	< 0.001	1.952	1.469–2.595	< 0.001
Male gender	0.856	0.647–1.133	0.278			
Age > 70	1.41	0.904–2.198	0.13			
T stage						
T1	Reference					
T2	0.644	0.260–1.595	0.341			
T3	0.829	0.378–1.815	0.639			
T4	0.795	0.371–1.705	0.556			
N stage						
N0	Reference					
N positive	0.888	0.737–1.069	0.209			
Location						
Right-sided colon	Reference					
Left-sided colon	1.387	0.917–2.097	0.121			
Rectum	1.425	0.986–2.059	0.059			
Lymph node examined	0.983	0.963–1.004	0.108			
Mucinous Adenocarcinoma	1.762	0.958–3.241	0.069			
Perineural invasion	1.124	0.826–1.528	0.457			
Vascular invasion	1.068	0.783–1.457	0.678			
CEA	1.557	1.168–2.075	0.003	1.346	1.003–1.808	0.048
Diabetes mellitus	1.074	0.676–1.706	0.763			
Obesity (BMI > 25)	1.269	0.909–1.770	0.162			

**Fig. 6:**
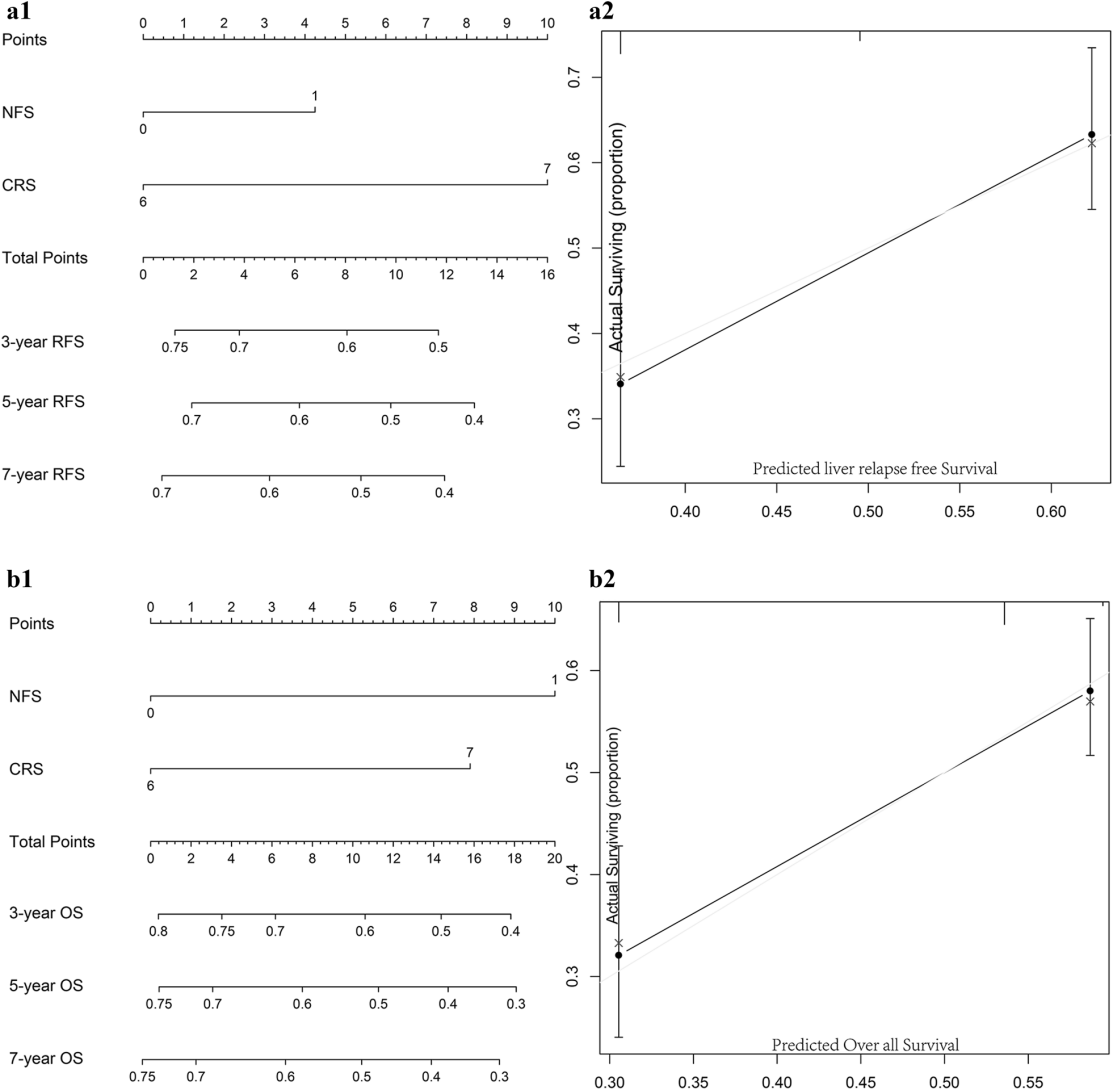
Construction of liver fibrosis score nomogram to estimate hepatic RFS and OS, along with the assessment of the model calibration. **a1** nomogram for liver RFS, **a2** Calibration curves for liver RFS. **b1** nomogram for OS, **b2** Calibration curves for OS

**Fig. 7:**
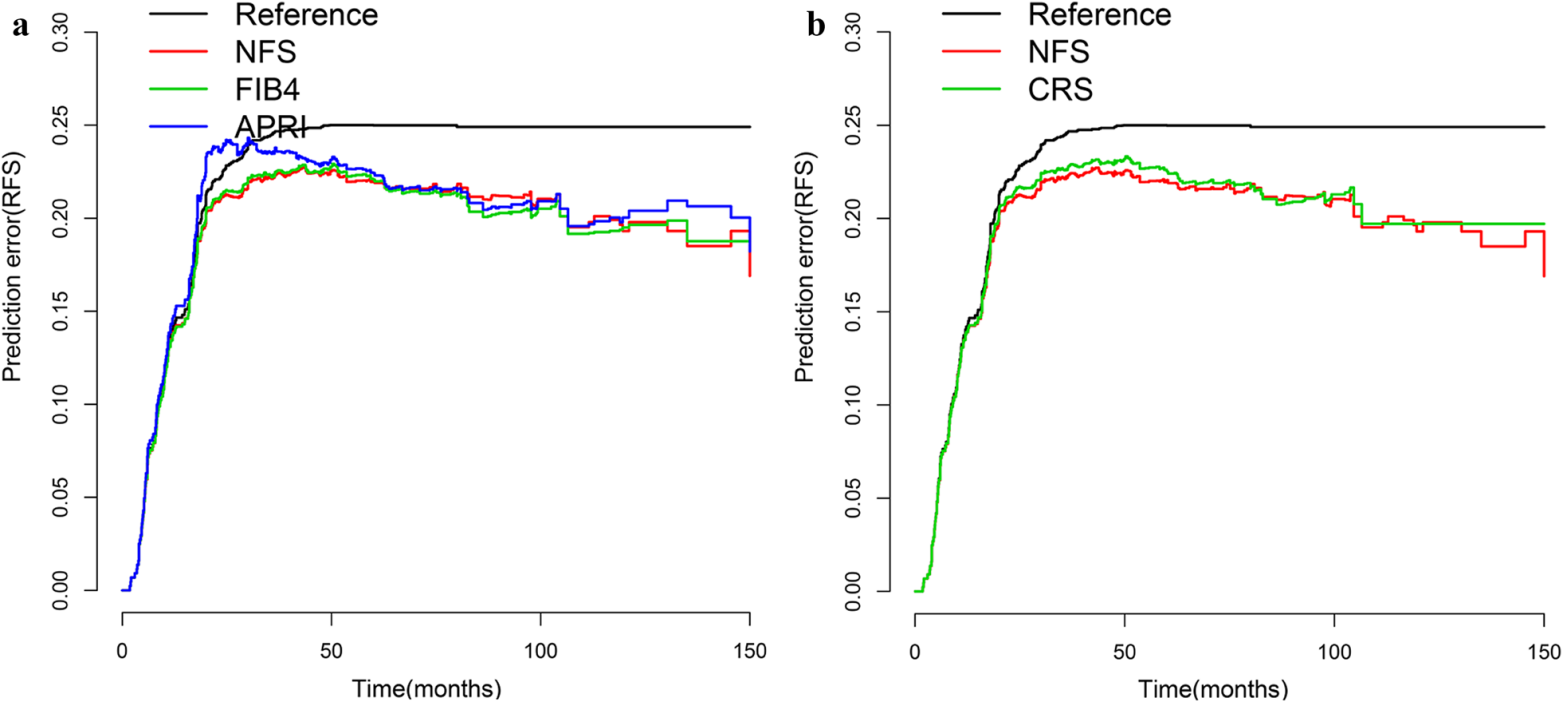
Prediction error curves for each model for stratifying liver RFS in the validation cohort. **a** Prediction error curves for the NFS, FIB4 and APRI score. **b** Prediction error curves for liver fibrosis and CRS score. Lower prediction errors indicate higher model accuracy

The CRS score by Fong was used to stratify patients in this cohort, the low-risk group had a 5-year hepatic RFS of 58.3%, which was higher than the rate of 36.6% in the high-risk group (P < 0.001). Hence, CRS score is the most popular and effective clinical risk score predicting RFS among patients undergoing hepatic treatment for colorectal liver metastasis. When liver fibrosis score incorporated outperformed the CRS score alone with respect to AUC and discriminatory ability (Fig. 6). AUC and Harrell’s C-index for the liver fibrosis score was 0.619 and 0.612 versus 0.596 and 0.596 for the CRS score (Additional file 1: Figure S10). The corresponding prediction error curves of models, in Fig. 7, showed that the liver fibrosis nomogram had a faintly lower error than the CRS score. Consistent with RFS results, the AUC and C-index were also higher in the liver fibrosis score compared with the CRS score, indicating better discriminatory ability.

## Discussion

To the best of our knowledge, this study firstly reported the prognostic significance of liver fibrosis in CRC following curative resection of primary colorectal tumors and hepatic metastasis. The results of this study demonstrated that patients with high liver fibrosis score developed much more liver metastasis and liver relapse compared to those with low liver fibrosis score. Competing risk regression showed the liver fibrosis was an independent prognostic factor for hepatic DFS, RFS and OS, even adjusting clinicopathologic features. Among these three noninvasive liver fibrosis scores, NFS score performed the best in predicting DFS, RFS and OS. What is more, liver fibrosis nomogram outperformed the CRS score by Fong [3]. The liver fibrosis score may challenge the existed prognostic power of clinical risk scores, and suggests a promising alternative.

The concordance study showed agreement among NFS, FIB4 and APRI. The NFS is estimated using a combination of six metabolic, inflammatory variables and clinical features. And FIB4 only considers patient age and ALT level. APRI does not include age. Hence, different results with the predefined cut-off values were achieved. In particular, distinctive racial features will affect the liver fibrosis definition, so the optimum cutoff values in Chinese were generated by the X-tile plots. NFS seemed to be stricter than FIB4 and APRI, and the clinical meaningfulness of the finding were compared. In fact, NFS, FIB4 and APRI were all shown to have the significantly positive predictive value in DFS, RFS and OS, in which NFS performed the best. The excellent performance of the NFS score might stem from its component variables. The NFS score includes age, diabetes/hyperglycemia, BMI (body mass index), platelet count, albumin, and AST/ALT ratio, which were all independent indicators of liver fibrosis. Hence, the prediction of liver fibrosis was correct in 90% [8]. This may explain the excellent performance of the NFS score predicting the survivals in liver metastatic CRC.

Above all, the liver fibrosis score is the first clinical risk score with organ-specific feature incorporated. Previous several known prognostic factors have been criticized for their reliance on primary demographic features, such as mucinous adenocarcinoma histology, perineural invasion and extramural venous invasion [12–14]. When the importance of metastatic organ-specific biology, seed and soil theory [15], is increasingly being recognized, the prognostic impact of many metastatic organ-specific markers has been evaluated [16, 17]. And our findings revealed that the noninvasive liver fibrosis scores could specifically predict liver metastasis and relapse. Our findings from this study have provided novel insight into the role of metastatic organ-specific microenvironments in progression of distant metastasis. There were several mechanisms accounting to the causation between fibrosis and hepatic metastasis. Firstly excessive deposition of connective tissue matrix in the liver may be one of the underling oncogenic factor, as suppression of hepatic fibrosis could reduce the occurrence of hepatic metastasis in murine model [18]. Secondly alternative mechanism for liver fibrosis enhancing liver metastasis formation may rely on cytokines release by hepatic stellate cells (HSC). HSC released several cytokines leading to hepatic fibrosis, meanwhile cross-talked with colon cancer cells and enhanced the invasiveness of cancer cells [19, 20]. HSC, vital in forming pre metastatic niches, can transdifferentiate from quiescence into greatly proliferative and mobile myofibroblasts [21]. The tumor associated extracellular matrix (ECM) was remodeled and deposited by active HSC, and will enhance migration and growth of metastatic cells [22–24]. In addition, the tumor angiogenesis niche was also promoted in liver fibrosis environment with multiple angiogenic factors, including VEGF, angiopoietin 1 and 2 [25, 26].

In contrast to the results of our study, when the liver fibrosis was evaluated by liver-to-spleen ratio (LSR) attenuation values in CT, Murono K [27] found hepatic metastases in CRC developed less frequently in patients with high fibrosis score. Considering NFS more sensitive and accurate than the LSR in terms of identifying liver fibrosis, that results derived from LSR might therefore underestimate the impact of liver fibrosis. Above all, our results obtained from the concordance analysis all demonstrated agreement among NFS, FIB4 and APRI. In addition, fatty liver in rats had been shown to be protective against liver metastasis in the liver metastatic models [28]. This discrepancy may be dependent on the cause and the degree of the steatotic liver, meanwhile other factors may also have influence, such as: species difference, tumor cells and injection methods. What is more, steatotic liver did not mean to develop fibrosis. Thus, there was the possibility that fibrotic progression was associated with the loss of the protective effect of steatosis on liver metastasis.

The liver fibrosis prognostic model also has some limitations. Firstly, its establishment was based on only single institution data. That is why the concordance analysis was performed and external validation in another cohort with the liver metastatic patients was conducted. Hence, this may suffice given the considerable large sample size and real-world data. Secondly, pathological findings are also helpful in providing clue for liver metastasis and relapse as it can demonstrate the immunological and histological aspects. However, our patients included stages I/II/III. It is ethically difficult to obtain normal liver tissue samples.

## Conclusions

This study demonstrated that CRC patients with liver fibrosis displayed significant risk of hepatic metastasis and relapse. In the meantime, NFS, FIB4 and APRI were used to identify an agreement in the concordance analysis. What is more, the fibrosis score model outperformed the most commonly used contemporary CRS score by the Fong. This current model offered numerous suggestions for crosstalk between tumor cells and specific organ niche, allowing to test some new targets and sequential stages.

## Supplementary information

**Additional file 1: Figure S1.** Liver fibrosis score in NFS, FIB4, APRI measured by Kaplan–Meier survival for OS in the discovery cohort. A1: original NFS score, A2: modified NFS score; B1: original FIB4 score, B2: modified FIB4 score; C: APRI score. **Figure S2.** Clinicopathologic factors measured by Kaplan–Meier survival for DFS and OS in the discovery cohort. **Figure S3.** TNM stage measured by Kaplan–Meier survival for DFS and OS in the discovery cohort, along with the assessment of AUC. **Figure S4.** Distribution of liver fibrosis score, liver metastasis and survival statuses in discovery cohort. **Figure S5.** The role liver fibrosis score in the liver metastasis stratified by TNM stage. A1: NFS in stage I + II, A2: NFS in stage III; B1: FIB4 in stage I + II, B2: FIB4 in stage III; C1: APRI in stage I + II, C2: APRI in stage III. **Figure S6.** Construction of liver fibrosis score nomogram to estimate OS in the discovery cohort, along with the assessment of the model calibration. A1: NFS nomogram, A2: Calibration curves for NFS; B1: FIB4 nomogram, B2: Calibration curves for FIB4; C1: APRI nomogram, C2: Calibration curves for APRI. **Figure S7.** Clinicopathologic nomograms for DFS and OS only using clinicopathologic risk factors in the discovery cohort, along with the assessment of the model calibration. A1: Clinicopathologic nomogram for DFS, A2: Calibration curves for DFS; B1: Clinicopathologic nomogram for OS, B2: Calibration curves for OS. **Figure S8.** Prediction error curves for each model for stratifying liver DFS and OS in the discovery cohort. A1: Prediction error curves of the NFS, FIB4 and APRI score for DFS. A2: Prediction error curves of liver fibrosis and Clinicopathologic nomogram for DFS. B1: Prediction error curves of the NFS, FIB4 and APRI score for OS. A2: Prediction error curves of liver fibrosis and Clinicopathologic nomogram for OS. Lower prediction errors indicate higher model accuracy. **Figure S9.** Nomograms for DFS and OS using conventional clinical metrics in the discovery cohort, along with the assessment of the model calibration. A1: conventional clinical metrics nomogram for DFS, A2: Calibration curves for DFS; B1: conventional clinical metrics nomogram for OS, B2: Calibration curves for OS. **Figure S10.** Liver fibrosis score and CRS measured by ROC curves for hepatic RFS and OS in the validation cohort. AUC: area under the curve; ROC: receiver operator characteristic. Liver fibrosis score outperformed the CRS.

## Data Availability

Not applicable.

## Abbreviations

CRC: Colorectal cancer
AUC: Area under the curve
OS: Overall survival
DFS: Disease free survival
RFS: Relapse free survival
NAFLD: Non-alcoholic fatty liver disease
NASH: Non-alcoholic steatohepatitis
FUSCC: Fudan University Shanghai Cancer Center
DM: Diabetes mellitus
HSC: Hepatic stellate cells
ECM: Extracellular matrix
