# Supplementary material for: Prediction of hepatic metastasis and relapse in colorectal cancers based on concordance analyses with liver fibrosis scores
Source: Clin Transl Med. 2020 Feb 5;9:13. doi: 10.1186/s40169-020-0264-3 (PMC7002812; doi:10.1186/s40169-020-0264-3)

Figure S1. Liver fibrosis score in NFS, FIB4, APRI measured by Kaplan-Meier survival for OS in the discovery cohort.

A1: original NFS score, A2: modified NFS score; B1: original FIB4 score, B2: modified FIB4 score; C: APRI score;

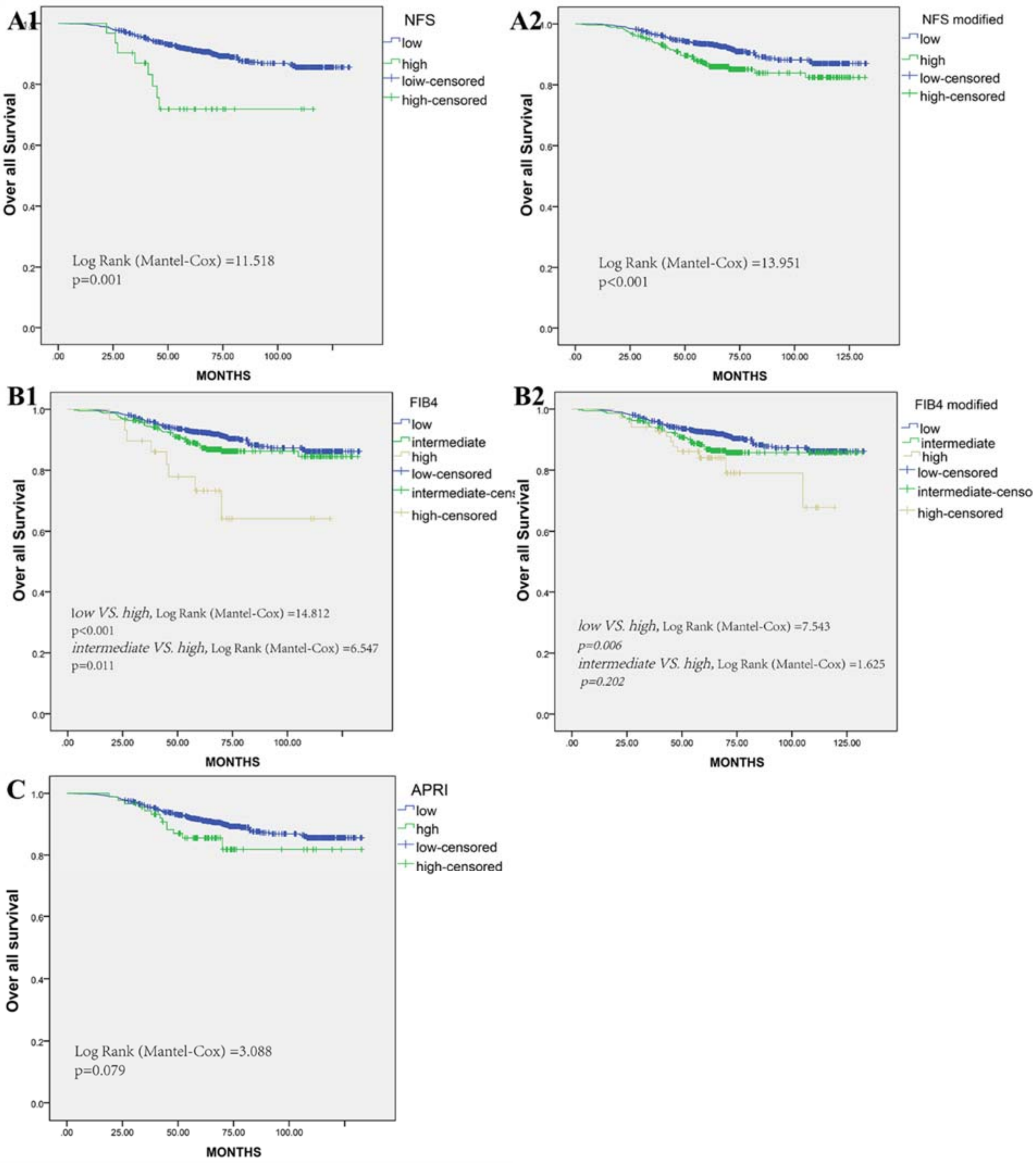

Figure S2. Clinicopathologic factors measured by Kaplan-Meier survival for DFS and OS in the discovery cohort.

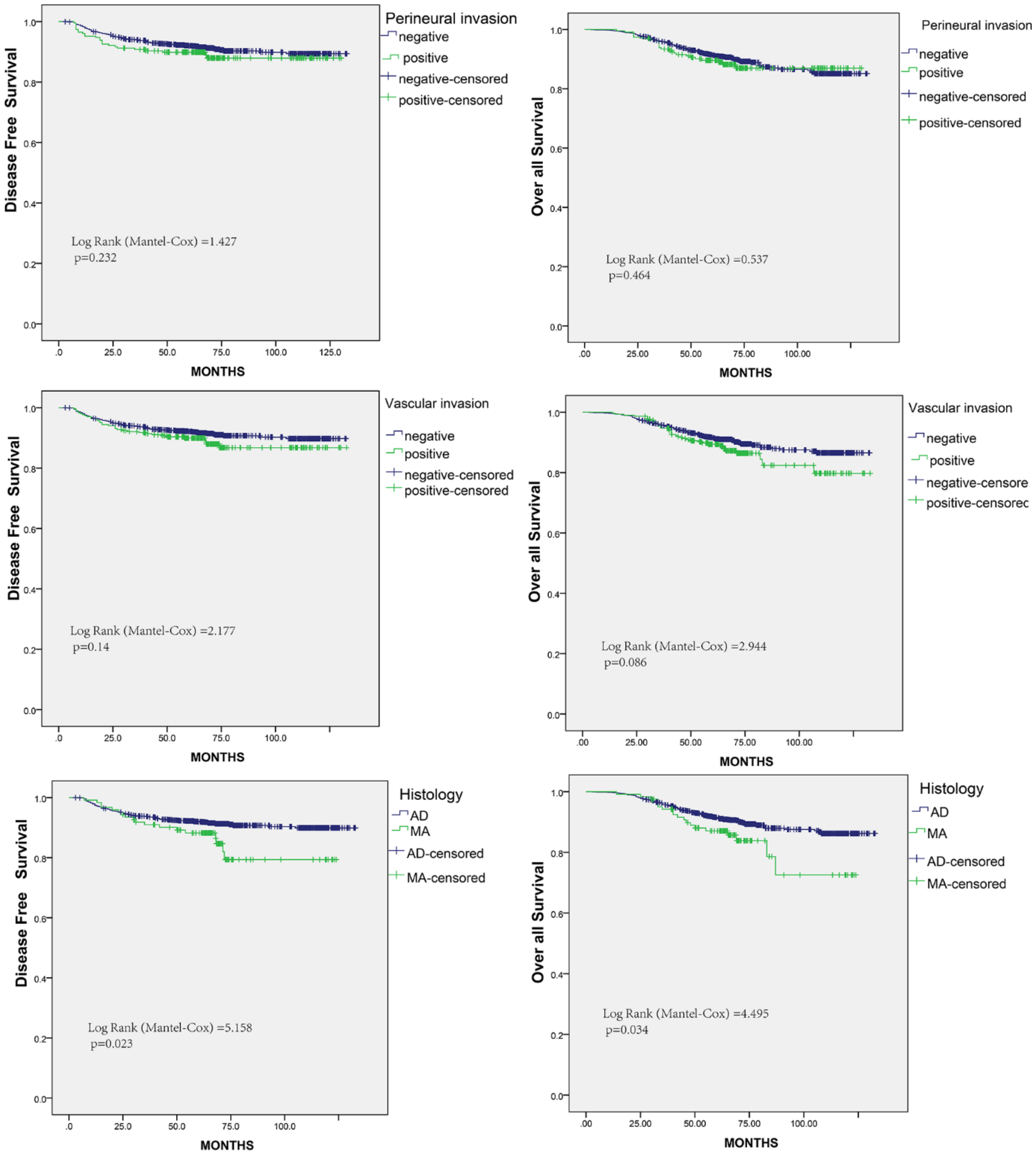

Figure S3. TNM stage measured by Kaplan-Meier survival for DFS and OS in the discovery cohort, along with the assessment of AUC.

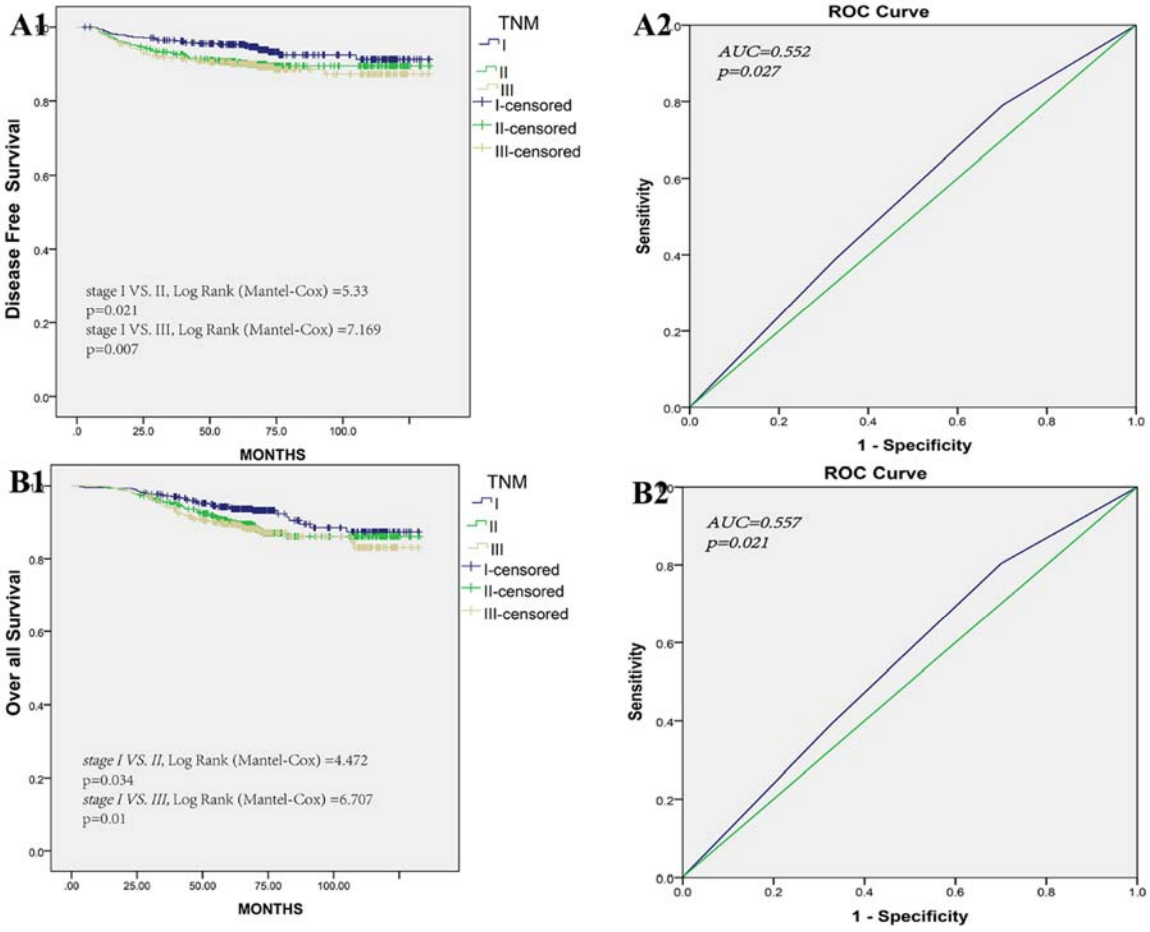

Figure S4. Distribution of liver fibrosis score, liver metastasis and survival statuses in discovery cohort.

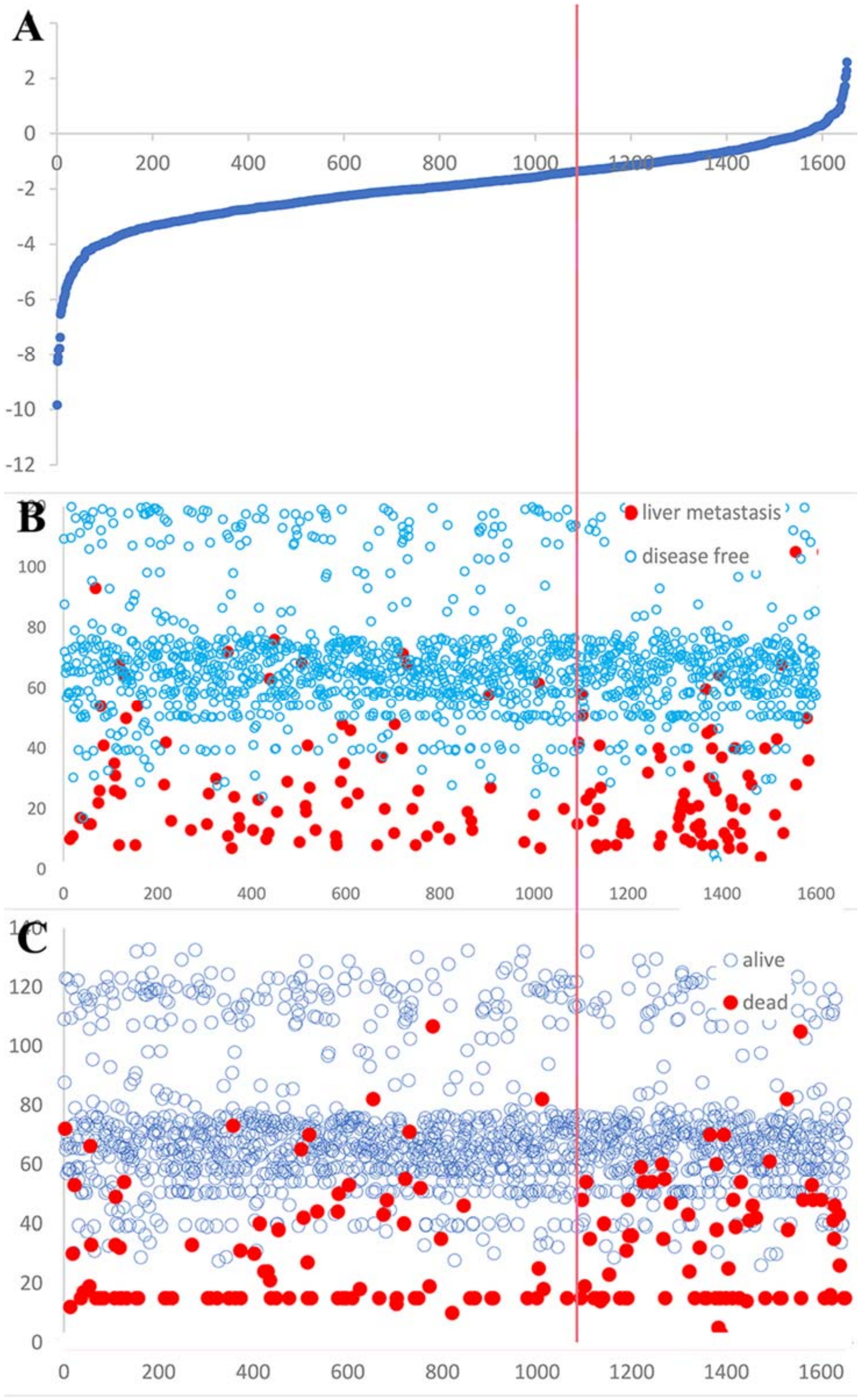

Figure S5. The role liver fibrosis score in the liver metastasis stratified by TNM stage.

A1: NFS in stage I + II, A2: NFS in stage III; B1: FIB4 in stage I + II, B2: FIB4 in stage III; C1: APRI in stage I + II, C2: APRI in stage III.

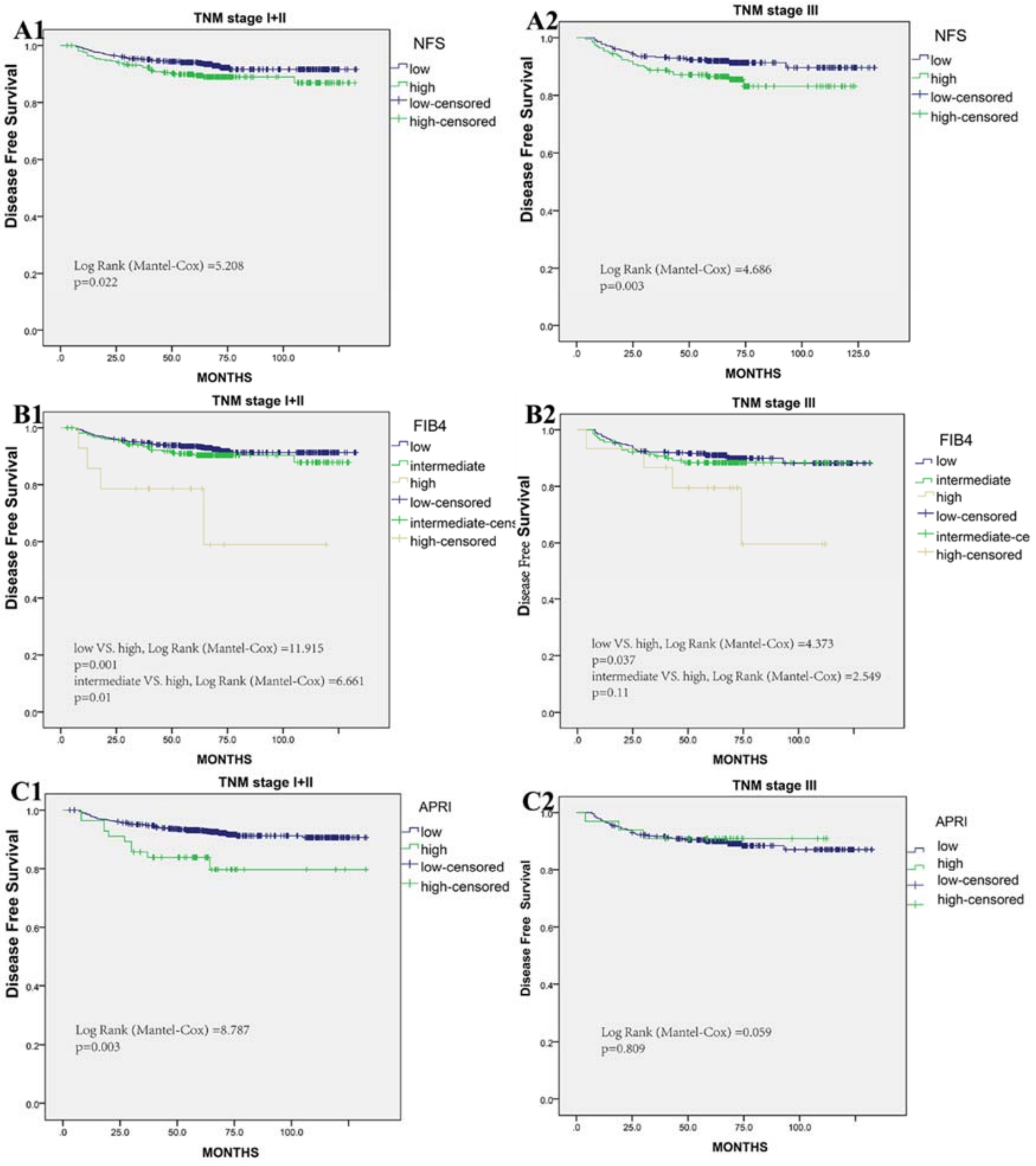

Figure S6. Construction of liver fibrosis score nomogram to estimate OS in the discovery cohort, along with the assessment of the model calibration.

A1: NFS nomogram, A2: Calibration curves for NFS; B1: FIB4 nomogram, B2: Calibration curves for FIB4; C1: APRI nomogram, C2: Calibration curves for APRI;

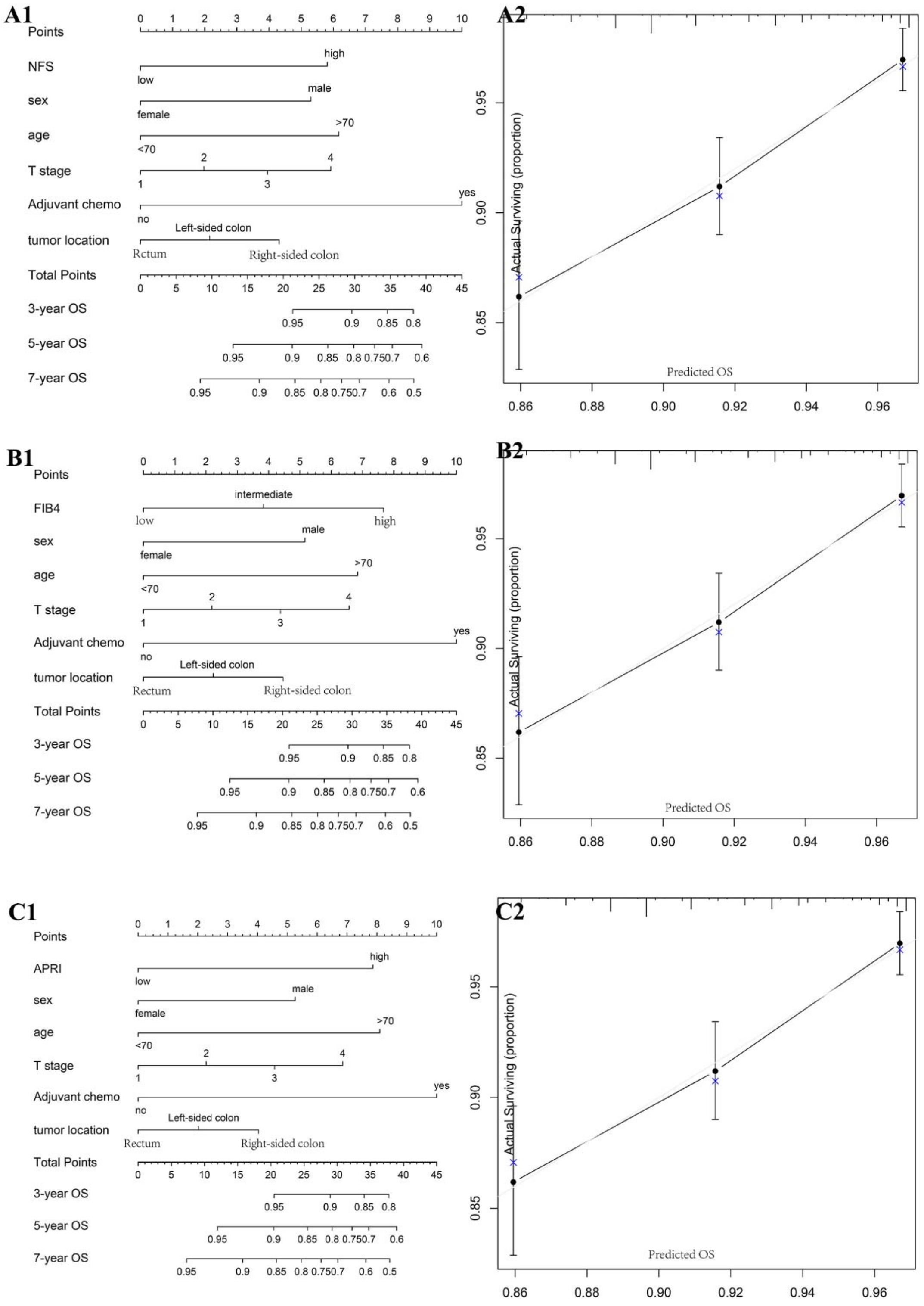

Figure S7. Clinicopathologic nomograms for DFS and OS only using clinicopathologic risk factors in the discovery cohort, along with the assessment of the model calibration.

A1: Clinicopathologic nomogram for DFS, A2: Calibration curves for DFS; B1: Clinicopathologic nomogram for OS, B2: Calibration curves for OS;

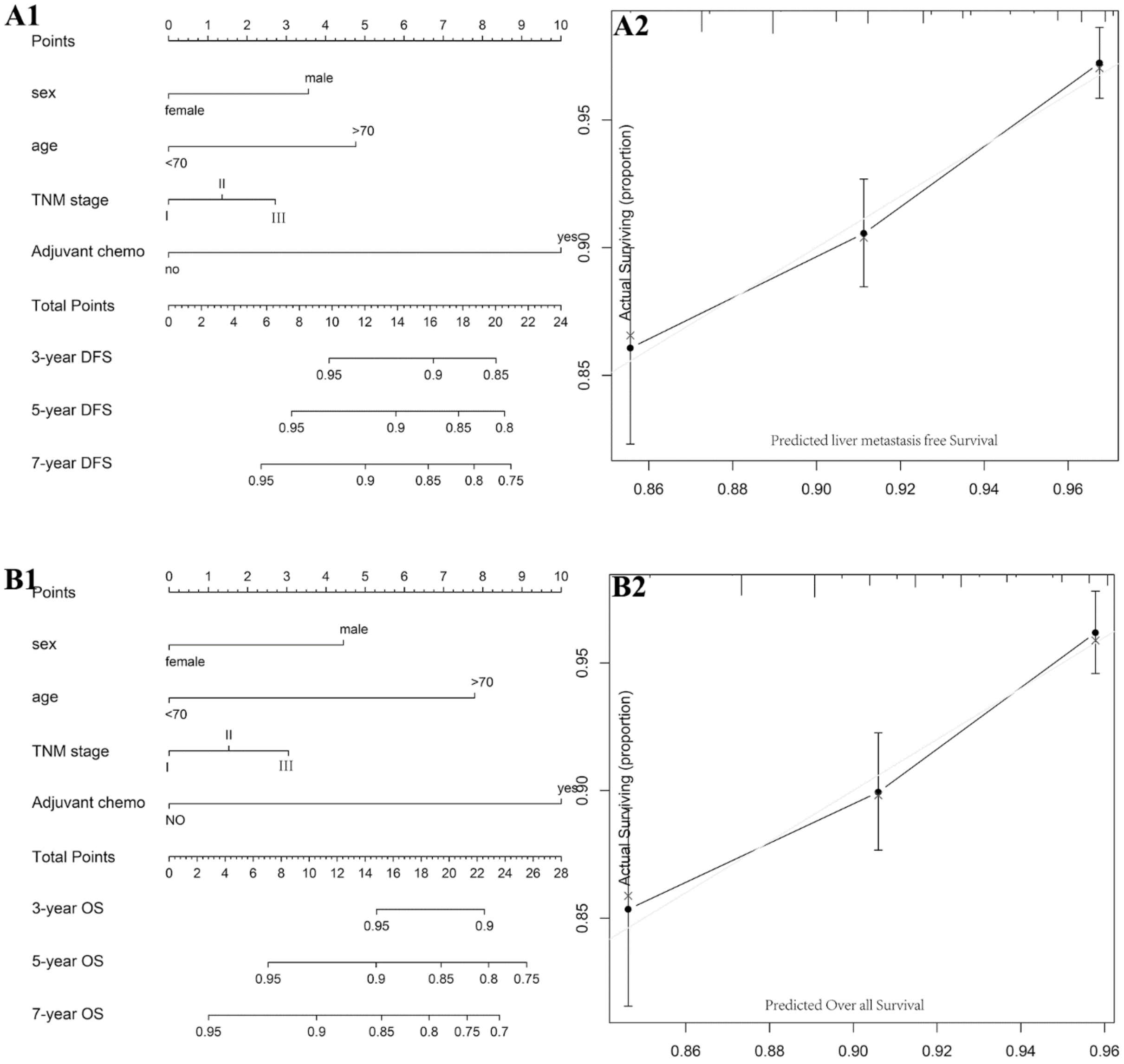

Figure S8. Prediction error curves for each model for stratifying liver DFS and OS in the discovery cohort.

A1: Prediction error curves of the NFS, FIB4 and APRI score for DFS. A2: Prediction error curves of liver fibrosis and Clinicopathologic nomogram for DFS. B1: Prediction error curves of the NFS, FIB4 and APRI score for OS. A2: Prediction error curves of liver fibrosis and Clinicopathologic nomogram for OS. Lower prediction errors indicate higher model accuracy.

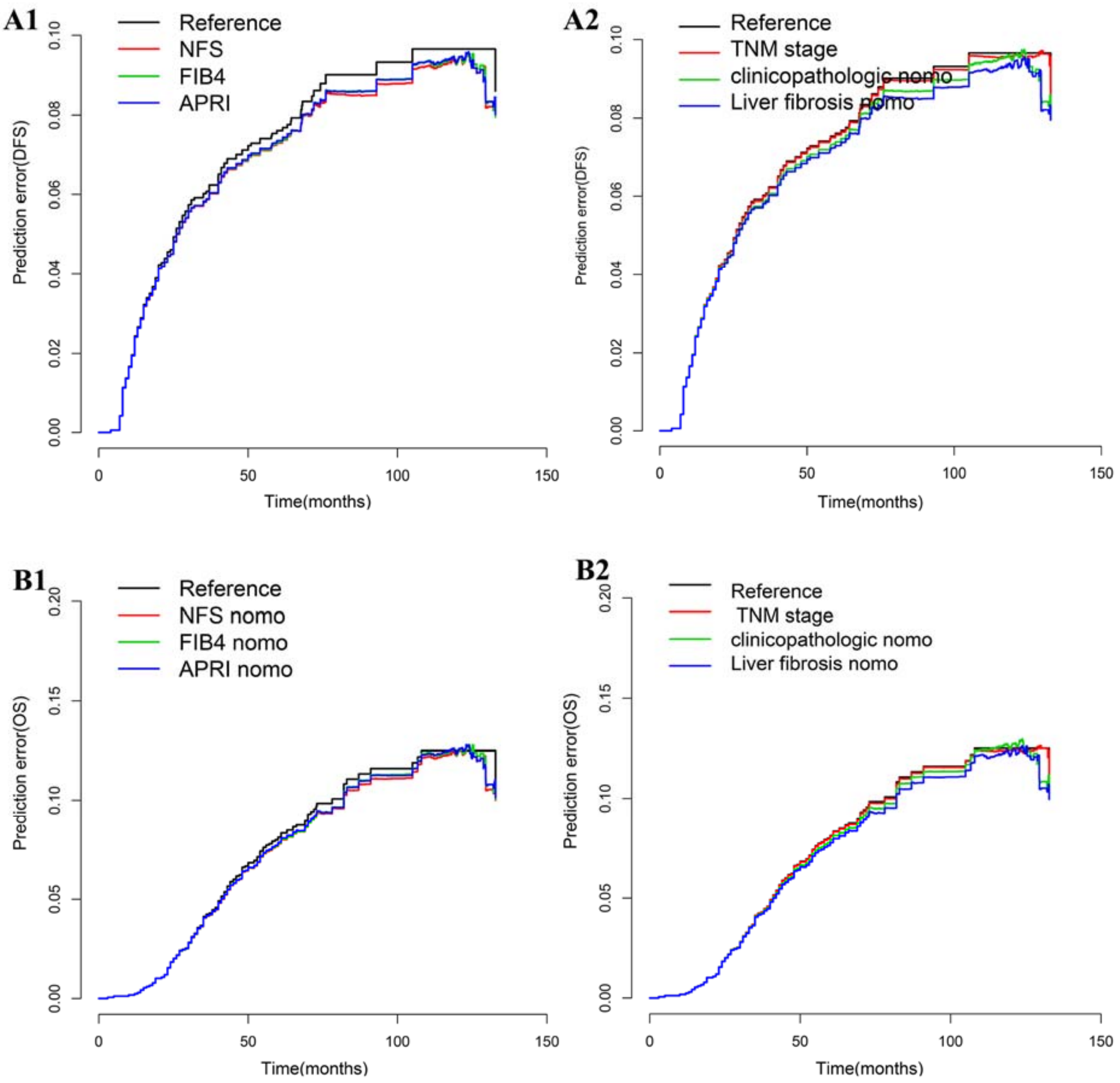

Figure S9. nomograms for DFS and OS using conventional clinical metrics in the discovery cohort, along with the assessment of the model calibration.

A1: conventional clinical metrics nomogram for DFS, A2: Calibration curves for DFS; B1: conventional clinical metrics nomogram for OS, B2: Calibration curves for OS;

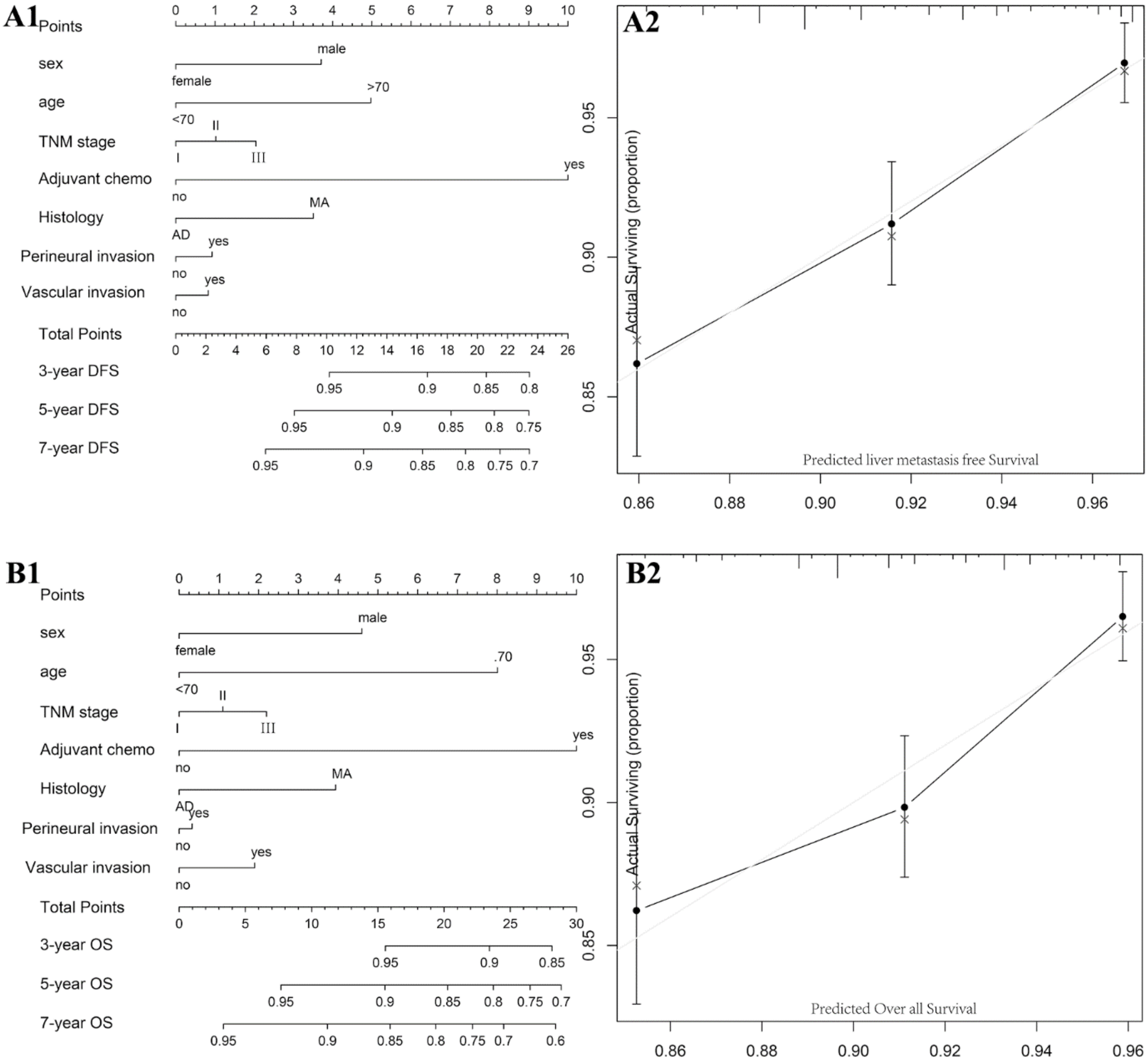

Figure S10. Liver fibrosis score and CRS measured by ROC curves for hepatic RFS and OS in the validation cohort.

AUC: area under the curve; ROC: receiver operator characteristic. Liver fibrosis score outperformed the CRS.

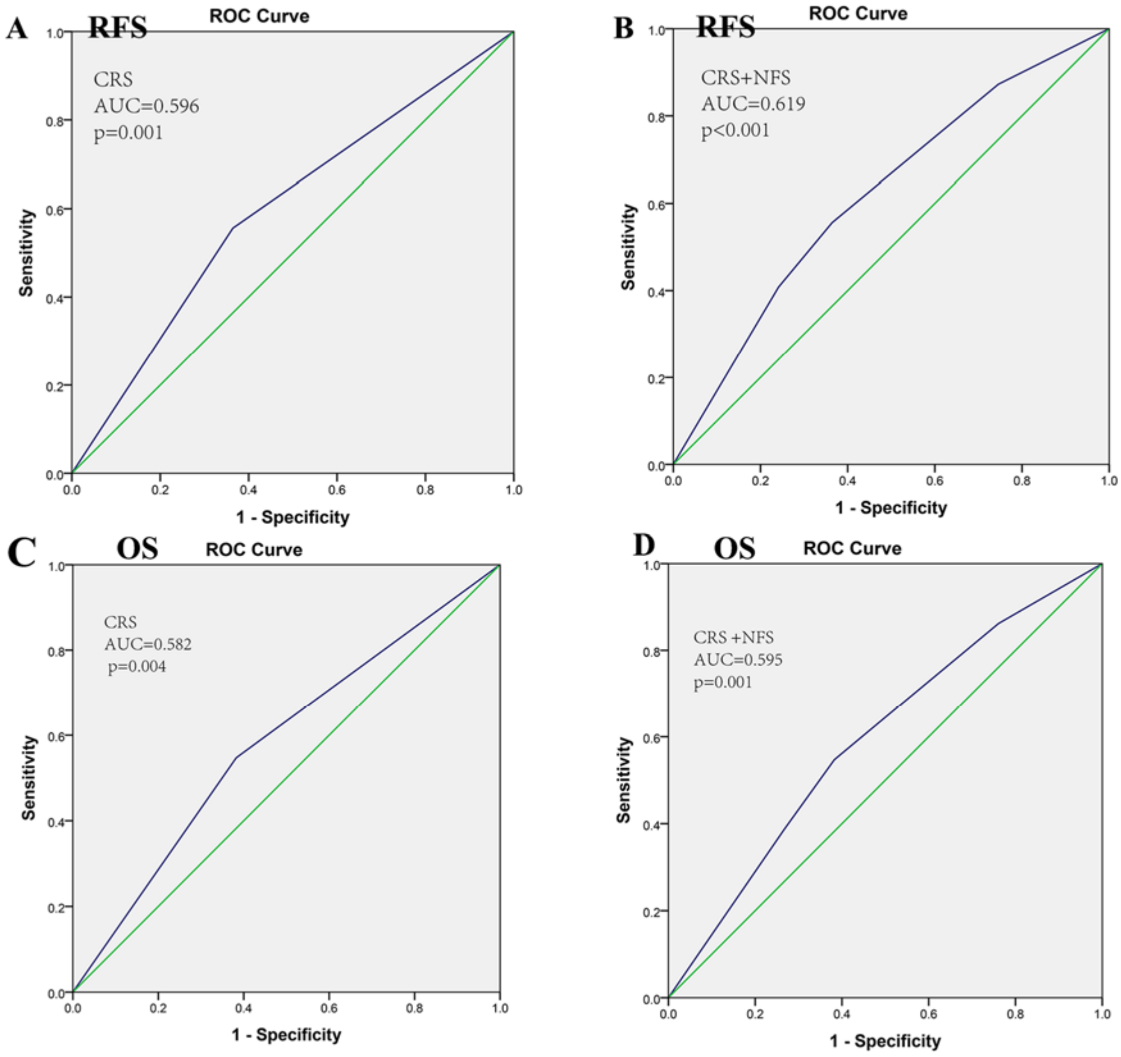

Supplement: Supplementary file 1 — Additional file 1: Figure S1. Liver fibrosis score in NFS, FIB4, APRI measured by Kaplan–Meier survival for OS in the discovery cohort. A1: original NFS score, A2: modified NFS score; B1: original FIB4 score, B2: modified FIB4 score; C: APRI score. Figure S2. Clinicopathologic factors measured by Kaplan–Meier survival for DFS and OS in the discovery cohort. Figure S3. TNM stage measured by Kaplan–Meier survival for DFS and OS in the discovery cohort, along with the assessment of AUC. Figure S4. Distribution of liver fibrosis score, liver metastasis and survival statuses in discovery cohort. Figure S5. The role liver fibrosis score in the liver metastasis stratified by TNM stage. A1: NFS in stage I + II, A2: NFS in stage III; B1: FIB4 in stage I + II, B2: FIB4 in stage III; C1: APRI in stage I + II, C2: APRI in stage III. Figure S6. Construction of liver fibrosis score nomogram to estimate OS in the discovery cohort, along with the assessment of the model calibration. A1: NFS nomogram, A2: Calibration curves for NFS; B1: FIB4 nomogram, B2: Calibration curves for FIB4; C1: APRI nomogram, C2: Calibration curves for APRI. Figure S7. Clinicopathologic nomograms for DFS and OS only using clinicopathologic risk factors in the discovery cohort, along with the assessment of the model calibration. A1: Clinicopathologic nomogram for DFS, A2: Calibration curves for DFS; B1: Clinicopathologic nomogram for OS, B2: Calibration curves for OS. Figure S8. Prediction error curves for each model for stratifying liver DFS and OS in the discovery cohort. A1: Prediction error curves of the NFS, FIB4 and APRI score for DFS. A2: Prediction error curves of liver fibrosis and Clinicopathologic nomogram for DFS. B1: Prediction error curves of the NFS, FIB4 and APRI score for OS. A2: Prediction error curves of liver fibrosis and Clinicopathologic nomogram for OS. Lower prediction errors indicate higher model accuracy. Figure S9. Nomograms for DFS and OS using conventional clinical metrics in th [file 40169_2020_264_MOESM1_ESM.pdf]
